# Symptom burden and surgical outcome in non-skull base meningiomas

**DOI:** 10.3389/fonc.2022.967420

**Published:** 2022-09-21

**Authors:** Tobias Mederer, Sebastian Schachinger, Katharina Rosengarth, Anja Brosig, Karl-Michael Schebesch, Christian Doenitz, Nils-Ole Schmidt, Martin Andreas Proescholdt

**Affiliations:** ^1^ Department of Neurosurgery, University Hospital Regensburg, Regensburg, Germany; ^2^ Wilhelm Sander-NeuroOncology Unit, University Hospital Regensburg, Regensburg, Germany; ^3^ Department of Otorhinolaryngology, University Hospital Regensburg, Regensburg, Germany

**Keywords:** meningioma, neurological deficit, resection, outcome, recurrence

## Abstract

**Purpose:**

Non-skull base meningiomas (NSBM) are a distinct entity and frequently present with focal neurological deficits. This study was designed to analyze functional and oncological outcome following microsurgical tumor resection in patients with NSBM.

**Patients and methods:**

An analysis of 300 patients that underwent NSBM resection between 2003 and 2013 was performed. Assessment measures for functional outcome were Karnofsky Performance Scale (KPS), Medical Research Council - Neurological Performance Scale (MRC-NPS), and improvement rates of focal deficits and seizures. The extent of resection; recurrence-free survival (RFS) and tumor-specific survival (TSS) were also determined.

**Results:**

Impaired KPS and MRC-NPS were present in 73.3% and 45.7%, respectively. Focal neurological deficits were recorded in 123 patients (41.0%), with hemiparesis (21.7%) and aphasia (9.3%) the most prevalent form of impairment. Most meningiomas were localized at the convexity (64.0%), followed by falcine tumors (20.3%). Both KPI and MRC-NPS scores were significantly improved by surgical resection. Postoperative improvement rates of 96.6%, 89.3%, 72.3%, 57.9%, and 27.3% were observed for aphasia, epilepsy, hemiparesis, cranial nerve, and visual field deficits, respectively. Long-term improvement was achieved in 83.2%, 89.3%, 80.0%, 68.4% and 54.6% of patients, respectively. Gross total resection (GTR) over subtotal resection (STR) significantly improved preoperative seizures and visual field deficits and correlated with reduced risk of new postoperative hemiparesis. Poor Simpson grade was the only significant prognostic factor in multivariate analysis for long-term functional deficit, which occurred in 7.3%. Median RFS was 45.9 months (6.0 - 151.5 months), while median TSS was 53.7 months (3.1 – 153.2 months). Both WHO grade (p= 0.001) and Simpson classification (p= 0.014 and p= 0.031) were independent significant prognostic factors for decreased RFS and TSS by multivariate analysis, respectively. Furthermore, tumor diameter > 50 mm (p= 0.039) significantly correlated with decreased TSS in multivariate analysis.

**Conclusion:**

Surgical resection significantly and stably improves neurological deficits in patients with NSBM.

## 1 Introduction

Meningiomas are the most frequent intracranial neoplasms and arise from arachnoid cap cells in the central nervous system (CNS) (1). According to the World Health Organization (WHO) classification of CNS tumors, meningiomas are divided into three grades with increasing malignancy (2). While roughly 80% of all meningiomas are WHO grade I with a good prognosis, the mortality and recurrence rates increase with WHO grades II and III (3). Based on tumor location, meningiomas are dichotomized into skull base (SBM) and non-skull base meningiomas (NSBM) (4). In addition to localization, several aspects indicate that SBM and NSBM are biologically and clinically distinct entities. Patients with NSBM present at an older age compared to SBM (5), and significantly more male patients are affected (6). Although a gross total resection defined as resection grade I or II according to the Simpson classification (7) is achieved more frequently (8, 9) and consequently, the recurrence rates are lower in NSBM (10), the progression-free interval is shorter (11) and the volumetric growth rate is significantly faster in NSBM (12). This aspect is reflected by the 2-4 times higher risk for WHO grade II or III malignancy grades (11, 13–16) and the significantly higher proliferation index (5, 17) in NSBM even when analyzing WHO grade I tumors only (9). The tendency of NSBM to develop more aggressive lesions may be caused by a different cell of origin in addition to a specific molecular framework of these tumors (18–20). Surgical resection in NSBM patients has three main goals: 1. Acquisition of tissue to establish a histological and molecular diagnosis (21); 2. Maximal removal of neoplastic tissue to achieve optimal tumor control (22); and 3. Decompression of eloquent brain - structures to normalize the neuro-functional status of the affected patients (23). Several studies have addressed the postoperative improvement of neurological symptoms after meningioma resection (24–30). However, no data are available reflecting the role of surgical resection on the functional status in NSBM patients as they reflect a separate entity with regard to localization, symptom burden, and clinical and biological dynamics. Therefore, our study aimed to assess the short and long-term impact of surgery on clinical performance scale rating, focal neurological impairments, and frequency of seizures as well as to evaluate prognostic factors for neurological improvement, tumor recurrence, and tumor-specific survival in NSBM patients.

## 2 Methods

### 2.1 Patient population and ethical approval

We conducted a prospective clinical registry for all patients diagnosed with an NSBM between 2003 and 2013 that underwent craniotomy and microsurgical tumor resection at the University Hospital of Regensburg. A total of 300 patients were included in this study. Skull-base meningiomas and patients under the age of 18 were excluded. Informed consent was obtained from all patients. A qualified staff neurosurgeon performed all tumor resections; the intraoperative findings were collected by reviewing the surgery reports. All data was either collected prospectively during follow-up appointments or retrospectively by reviewing outpatient records and/or by contacting the patient’s primary care physician. The study was conducted in accordance with the ethical standards of the Helsinki Declaration and approved by the local ethics review board (20-1799-101).

### 2.2 Functional assessment

Clinical, neurological and oncological outcome was evaluated at three time points: preoperative, postoperative, and last follow-up. Clinical and neurological performance was classified by the Karnofsky Performance Scale (KPS) and the Medical Research Council - Neurological Performance Scale (MRC-NPS) (31). Tumor recurrence was classified as progression of residual tumor or tumor recurrence after gross total resection (GTR) in follow-up brain imaging according to RANO criteria (32).

### 2.3 Imaging analysis

Patients received preoperative MRI scans according to a standard screening protocol including in T1-weighted imaging with and without contrast agent, T2-weighted-, FLAIR and diffusion-weighted imaging. Lesions that showed more than 35% peritumoral FLAIR or T2 hyperintensity in relation to the tumor volume were classified as tumors with significant perifocal edema. The largest axial diameter in T1-weighted, contrast-enhanced imaging was measured for tumor size assessment. On the day after surgery, patients underwent a postoperative CT scan. Follow-up imaging included a baseline MRI 3 months after surgery, followed by yearly MRI scans in grade I meningiomas. Higher grade meningiomas were scanned every 6 (grade II) and 3 months (grade III). Extent of resection (EOR) was evaluated by reviewing surgical reports and by an independent neuro-radiologist based on the postoperative baseline MRI scans.

### 2.4 Histopathological assessment

Histopathological diagnoses were performed by independent neuropathologists according to the WHO grading system for meningiomas. MIB-1 labeling index was determined by neuropathologists as the percent of positively stained tumor cell nuclei in a minimum of four high magnification (400x) visual fields.

### 2.4 Statistical analysis

Descriptive and comparative statistical analyses were performed with Stata software (version 14.2, Stata Corporation, College Station, TX, USA). Continuous variables are reported as mean, median, and range. Rates and proportions were analyzed using Chi square analysis, group differences were detected by performing two-tailed Mann-Whitney testing, and one-way repeated measure ANOVA. To analyze survival rates, the Kaplan-Meier method was applied, univariate analysis was performed by log-rank test, and multivariate testing was performed by calculating a multivariate logistic regression or a multivariate Cox regression analysis. Violin plots and Sankey plots were created with the online software PlotsOfData (33) and RAWGraphs (34) and modified with Adobe Illustrator CC 2018.

## 3 Results

### 3.1 Description of patient characteristics and treatment pattern

**Figure 1:**
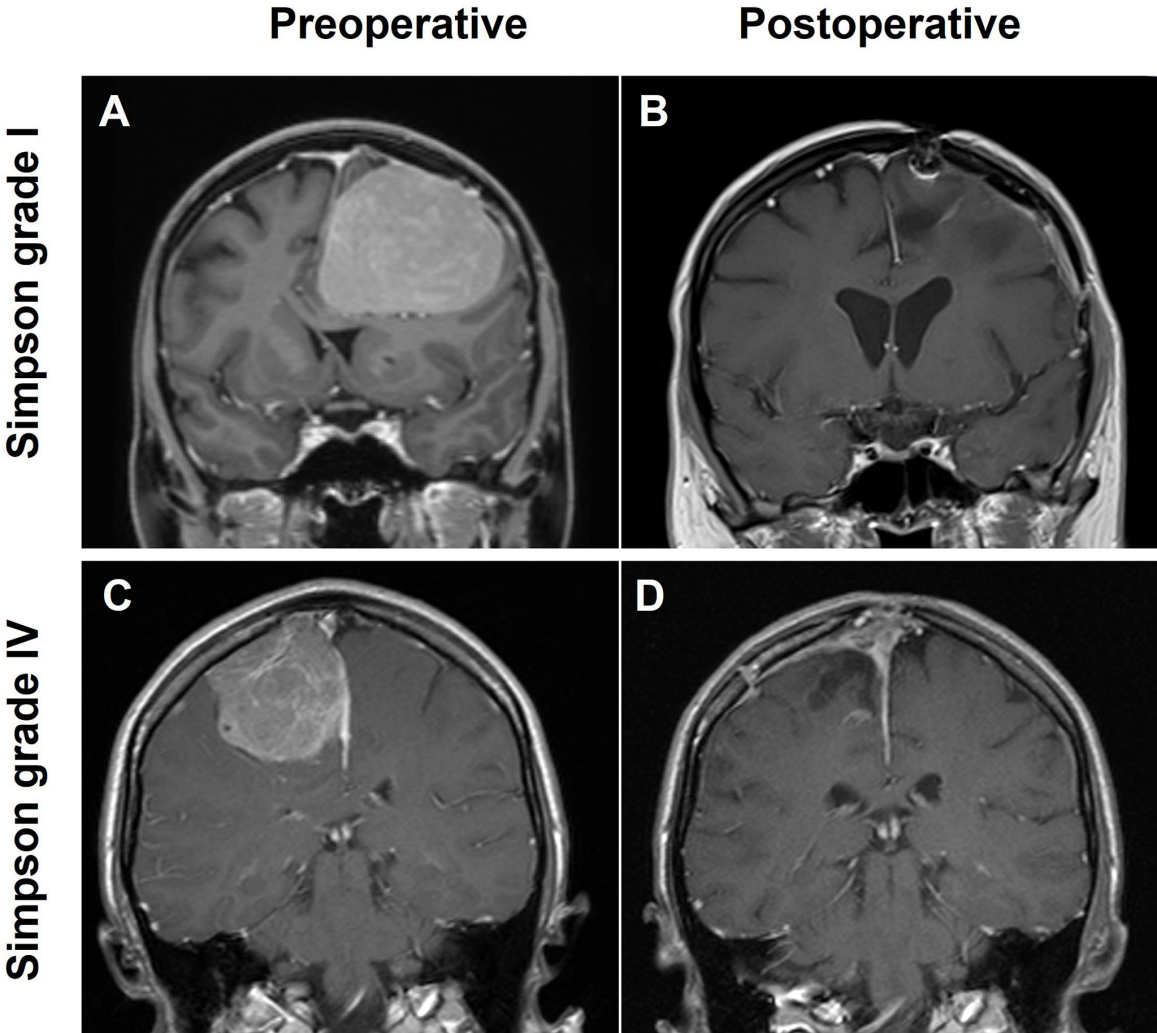
Illustration of two cases with large NSBM; T1-weighted, contrast-enhanced MRI scans in coronal orientation are shown. **(A)** Convexity meningioma receiving a grade Simpson I resection **(B)**, **(C)** a parasagittal lesion with a Simpson IV resection **(D)**.

A total of 300 consecutive patients with an NSBM surgically treated at the University Hospital of Regensburg between 2003 and 2013 were included in this study. The majority of resections (95.7%) were either performed or supervised by a team of 5 board-certified attending neurosurgeons with a comparable level of experience. The clinical baseline characteristics of the entire study population are summarized in **Table 1**. The median age was 60.6 years (range: 25.2 - 89.1 years) with a male-to-female ratio of 1:2.3 (91 males and 209 females). The median follow-up time was 87.0 months (range: 3 - 153.4 months). Histopathological diagnosis showed 253 (84.3%) WHO I, 44 (14.7%) WHO II, and 3 (1.0%) anaplastic WHO III meningiomas. Most tumors were localized at the convexity (64.0%), followed by falcine tumors (20.3%), from which the anterior third of the falx was most prevalently affected (68.8%). Parasagittal tumors occurred in 15.7% of all patients. The predominant brain lobe locations were frontal and fronto-parietal with 47.3% and 23.7%, respectively. Gross total resection (GTR) corresponding to Simpson I (51.0%) and Simpson II (28.3%) was achieved in 79.3% of the patients. In 62 patients (20.7%) only subtotal resection (STR) could be achieved (Simpson III: 7.0%, Simpson IV: 13.3% and Simpson V: 3.3%; **Figures 1A–D**). We found a significantly worse GTR rate in parasagittal tumors (38.3% GTR vs. 86.89% and 86.98% in falcine and convexity, respectively; p =0.0001). A total of 16 patients (5.3%) received radiation treatment. Immediately after resection, seven patients were treated with radiation (2.3%; 6 WHO grade II tumors and 1 WHO grade III tumor). Four patients (1.3%) were radiated following resection of a recurrent tumor, and two patients with recurrent tumors received radiation without another resection. Finally, three patients received radiation after the second recurrence. In incomplete resections, the decision for radiation treatment was made based on clinical, radiological, and histological criteria in the interdisciplinary neurooncological tumor board. In the majority of cases with incomplete resection of a WHO grade I tumor, radiation treatment was started whenever signs of tumor progress were detected.

**Table 1:** Clinical characteristics of the entire patient cohort.

Variable	Number (%)
N =	300
**Age (years)**	60.6
Median Range	(25.2 – 89.1)
**Sex (f/m)**	209 (69.7)/91 (30.3)
**Follow up time (months)**	87
Median Range	3 – 153
**WHO grade**	253 (84.3)
I II III	44 (14.7) 3 (1.0)
**MIB-1 labeling index (%)**	5.3
Mean	
**Tumor diameter (mm)**	
Median Range	37 4-120
**Presurgical KPI**	
Median Range	90 60 - 100
**Extent of resection**	
GTR (Gross total resection) STR (Subtotal resection)	238 (79.3) 62 (20.7)
**Simpson classification**	
I II III IV V	153 (51.0) 85 (28.3) 21 (7.0) 40 (13.3) 1 (0.3)
**Bone infiltration**	
Yes No	75 (25.0) 225 (75.0)
**Venous sinus infiltration**	
Yes No	72 (24.0) 228 (76.0)
**Localization**	
Convexity Falx cerebri Parafalcine	192 (64.0) 61 (20.3) 47 (15.7)
**Side**	
Left Right Bilateral	147 (49.0) 136 (45.3) 17 (5.7)
**Lobe**	
Frontal Fronto-parietal Parietal Parieto-occipital Occipital Temporal Fronto-temporal Temporo-parietal Temporo-occipital	142 (47.3) 71 (23.7) 31(10.3) 16 (5.3) 14 (4.7) 14 (4.7) 8 (2.6) 3 (1.0) 1 (0.3)

While 89.7% of the patients initially presented with tumor-related symptoms, 34 of the patients (10.3%) were diagnosed with meningioma because of non-related symptoms that had led to brain imaging. The most frequent presenting symptoms for NSBM consisted of headache (32.3%), generalized or partial seizures (29.7%), and hemiparesis (17.3%). Aphasia or visual field deficits were seen in 9.3% and 3.7% of the patients, respectively. Hemiparesis occurred significantly more frequently in frontoparietal tumors compared to all other affected lobes (p = 0.027). Visual field deficits were more prevalent in parasagittal tumors (p = 0.010). Psychoorganic syndrome (memory loss, emotional lability, reduced intellectual capacity) was significantly more frequent in falcine tumors with 13.1% compared to 6.4% in parasagittal and 3.65% in convexity tumors (p = 0.025). Seizures occurred significantly more frequently in convexity tumors (27.08%) vs. 13.11% and 10.64% in falcine and parasagittal tumors, respectively (p = 0.009). WHO grades and histology classes were evenly distributed throughout the lobes and the location of the tumors. Tumors with a higher grade of malignancy (WHO grade II&III) presented significantly more frequently with large perifocal edema compared to WHO grade I tumors (59.9% vs. 38.1%; p = 0.012)). Presurgical median KPI and NPS were significantly worse in WHO grade II&III tumors compared to WHO grade I tumors (p = 0.006 and 0.0156, respectively), also, focal neurological deficits were significantly more frequent in patients with grade II&III compared to grade I tumors (p = 0.001).

### 3.2 Surgical morbidity and mortality

Perioperative complications were seen in 62 patients (20.7%), while 87.1% of these patients had pre-consisting comorbidities such as arterial hypertension (38.3%), thyroid disease (15.3%), diabetes (13.0%), other neoplasms (10.0%), obesity (9.3%), coronary heart disease (4.0%), smoking (3.6%) and chronic obstructive pulmonary disease (3.3%). In addition, patients showing postoperative complications were significantly older compared to those without complications (p = 0.030). The most frequent complications were CSF leaks (10.6%), wound healing disorders (7.3%), and intracranial hemorrhage (4.3%) (**Table 2**). The mortality rate was 1.0%, while the three patients that died within 30 days of surgery had either low preoperative Karnofsky Performance Scores (50-60) or higher-grade meningioma (WHO II).

**Table 2:** Postoperative complications.

Complication	Number (%)
**CSF leakage**	32 (10.6)
**Wound healing disorder**	22 (7.3)
**Intracranial hematoma**	13 (4.3)
**Pulmonary embilism**	8 (2.7)
**Increased ICP**	6 (2.0)
**Stroke**	4 (1.3)
**Pneumonia**	3 (1.0)
**Cardiac complications**	1 (0.3)
**Sinus vein thrombosis**	1 (0.3)

### 3.3 Functional outcome

**Figure 2:**
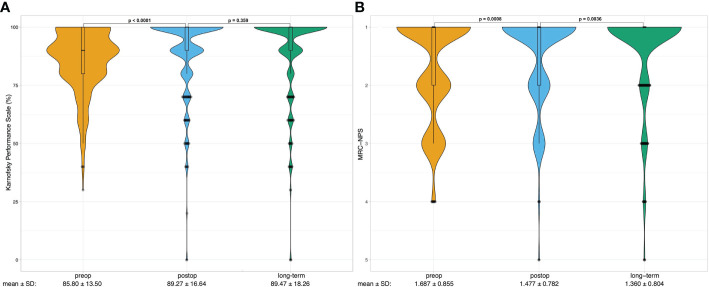
Violin plots show the distribution of preoperative (preop, yellow), postoperative (postop, blue) long-term (last follow-up, green) **(A)** Karnofsky Performance Scale (KPS) scores and **(B)** Medical Research Council - Neurological Performance Scale scores of the analyzed patients (n=300). Boxplots are shown within violin plots depicting median with lower and upper quartiles. Whiskers represent 1.5 interquartile range. Outliers are depicted as points. One-way repeated measure ANOVA was calculated to analyze the difference between preop, postop and long-term performance, the p – values are noted on top of the graph. Below the plots mean scores with standard deviations (SD) are shown.

The functional outcome of the patients following craniotomy and microsurgical tumor resection was assessed by the Karnofsky Performance Status (KPS) and the Medical Research Council - Neurological Performance Scale (MRC-NPS) preoperatively, postoperatively, and during the long-term follow-up. Both KPS and MRC-NPS scores improved significantly upon surgery (85.80 vs. 89.27, p < 0.0001 and 1.687 vs. 1.477, p = 0.0008 respectively) (**Figures 2A, B**). During the follow-up the KPS remained stable (89.47, P = 0.359), whereas the neurological outcome - measured by the MRC-NPS - further improved (1.360, p = 0.0036) (**Figure 2B**). Patients with grade I tumors showed significantly more frequent improvement of both presurgical KPS and MRC-NPS compared to patients with grade II&III tumors (p = 0.004 and p = 0.026, respectively). No significant differences in the KPS or MRC-NPS improvement rates were detected between the separate tumor locations.

#### 3.3.1 Epilepsy

**Figure 3:**
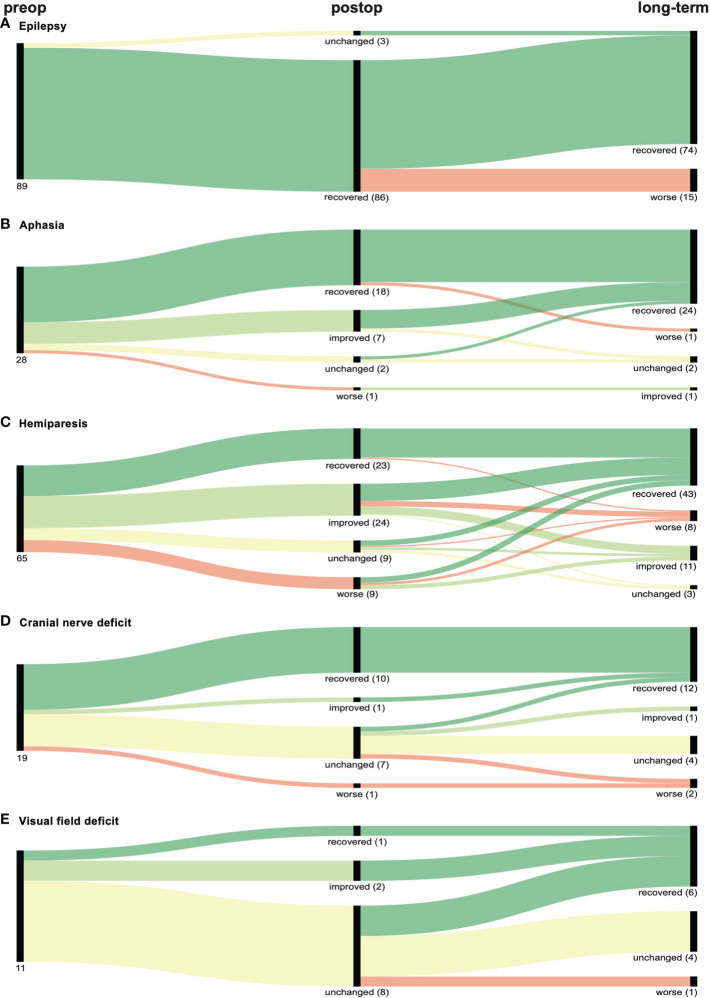
Graphical representation (Sankey plots) of the neurological outcome of patients with preoperative (preop) **(A)** epilepsy, **(B)** aphasia, **(C)** hemiparesis, **(D)** cranial nerve deficit or **(E)** visual field deficit within 30 days after surgery (postop) and at last follow-up (long-term). Symptom improvement and recovery is represented in light and dark green respectively while worsening of symptoms is depicted in red. No change in deficits is marked yellow. Numbers in parenthesis indicate number of patients of each branch.

86 of the 89 patients (96.6%) presenting with epilepsy preoperatively were free of seizures following the surgery, and 71 (79.8%) patients remained stable during the follow-up. While for 15 patients that had improved after tumor resection, epilepsy re-occurred, three patients showing no neurological improvement upon surgery were asymptomatic in the follow-up, resulting in a long-term improvement rate of 83.2% (**Figure 3A**).

#### 3.3.2 Aphasia

Aphasia improved both postoperatively and long-term in 25 of the 28 patients (89.3%). One patient experienced worsening of aphasia after being asymptomatic following surgery, while another patient improved during the follow-up (**Figure 3B**).

#### 3.3.3 Hemiparesis

For hemiparesis, we observed an improvement rate of 72.3% (47/65), while 64.6% (42/65) remained stable in the follow-up. Interestingly, 44.4% (4/9) of patients with initial worsening completely regained motor function in the follow-up, resulting in an overall long-term improvement rate of 80.0% (52/65) (**Figure 3C**).

#### 3.3.4 Cranial nerve deficits

Cranial nerve deficits were seen in 19 preoperative patients, of which 57.9% (10/19) and 68.4% (13/19) improved after surgery and in the follow-up, respectively. Two patients (10.6%) experienced postoperative worsening of the deficit. (**Figure 3D**).

#### 3.3.5 Visual field deficits

The lowest improvement rates were observed for visual field deficits. Three of eleven patients (27.3%) improved postoperatively, while a total of six patients (54.6%) improved in the follow-up. One patient (9.1%) experienced exacerbated visual field deficit after tumor resection (**Figure 3E**).

#### 3.3.6 Neurological morbidity

A total of 43 patients (14.3%) experienced a new neurological deficit directly following NSBM surgery. This postoperative neurological morbidity was 9.7%, 4.3%, 2.3%, and 0.7% for newly occurring hemiparesis, epilepsy, aphasia and cranial nerve deficits, respectively. No new postoperative visual field deficit was observed. The deficit remained unchanged in 34.8% (8/23) for hemiparesis, 38.5% (5/13) for epilepsy, and 42.9% (3/7) for aphasia. Thus, 13 patients experienced a new permanent neurological deficit or worsening of a pre-consisting deficit, resulting in an overall neurological morbidity of 4.3% at follow-up. When combining neurological morbidity with reduced clinical performance after tumor resection measured by long-term KPS scores, 22 patients (7.3%) showed long-term neurological or clinical deterioration following surgery for NSBM. Univariate analysis revealed a significant correlation between WHO grading (p = 0.001), MIB labeling index (p = 0.002), Simpson classification (p = 0.002), venous sinus infiltration (p = 0.003) and tumor diameter ≤/> 50 mm (p = 0.042) with the long-term functional outcome. Age, sex, localization, and bone infiltration did not significantly correlate with permanent neurological or clinical deterioration. Upon multivariate logistic regression analysis, only poor Simpson grade remained a significant independent prognostic factor for decreased functional outcome (p = 0.012).

#### 3.3.7 Effect of extent of resection on functional recovery

The extent of resection (EOR) was not associated with the postoperative improvement rates of the presurgical KPS and MRC-NPS scores (p = 0.122 and p = 0.365, respectively). However, seizures and visual field deficits were more likely to improve postoperatively when GTR of the tumor was achieved (p = 0.041 and 0.026, respectively). No significant differences were found between GTR and STR in the improvement of hemiparesis (p = 0.869), aphasia (p = 0.435), and cranial nerve deficits (p = 0.570) (**Table 3**). There was no significant difference in the incidence of new postoperative deficits between GTR versus STR, except for hemiparesis. A new postoperative hemiparesis was less likely to occur when GTR was achieved (p = 0.013) (**Table 3**).

**Table 3:** Improvement and worsening rates of seizures and focal neurological deficits stratified by gross total resection (GTR) vs. subtotal resection (STR) in NSBM patients.

Deficit	EOR^*^	Postoperative change			
		Improvement rate (%)	p-value	Worsening rate (%)	p-value
**Seizures**	GTR	89.3	0.041	9.7	0.522
	STR	69.2		3.3	
**Aphasia**	GTR	91.3	0.459	2.5	0.759
	STR	80.0		3.2	
**Hemiparesis**	GTR	74.5	0.529	8.4	0.013
	STR	66.6		19.4	
**Cranial nerve deficit**	GTR	52.9	0.202	1.3	0.374
	STR	84.4		0.0	
**Visual field deficit**	GTR	60.0	0.026	0.0	NA^**^
	STR	0.0		0.0	

*EOR, Extent of resection.

**NA, Not applicable.

### 3.4 Survival outcome

#### 3.4.1 Recurrence-free survival outcome

**Figure 4:**
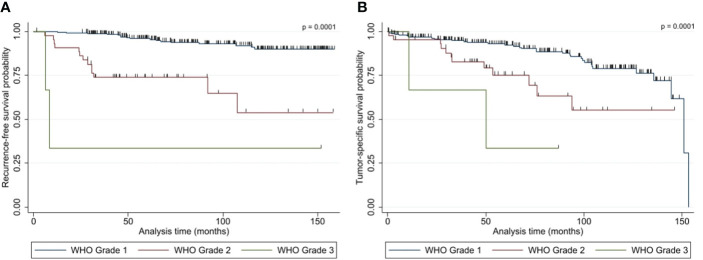
Kaplan-Meier plots show the **(A)** recurrence-free survival and **(B)** overall survival of patients with NSBM following tumor resection based on WHO grading (n=300). P-values (log rank test) are shown.

Within the follow-up, 42 (24 female and 18 male) patients (14.0%) presented with a tumor recurrence. 54.8% (23/42) of the recurred tumors were WHO I, while 40.5% (17/42) and 4.8% (2/42) were WHO II and WHO III. The median RFS was 45.9 months (6.0 - 151.5 months). The Kaplan-Meier plot for the RFS stratified by WHO grades are shown in **Figure 4A**. The recurrence-rates were 9.1%, 38.6%, and 66.7% for WHO I, II, and III meningiomas, respectively. Univariate analysis (log-rank testing) of tumor characteristics showed a significant correlation of WHO grade (p = 0.0001), Simpson classification (p = 0.0040), venous sinus infiltration (p = 0.0010) and tumor diameter ≤/> 50 mm (p = 0.0250) with RFS (**Table 4**). The MIB labeling index, age, sex, localization, and bone infiltration did not significantly correlate with RFS (p > 0.05). Upon multivariate logistic regression analysis of the significant variables in the univariate testing, the WHO grade (p = 0.0001), the Simpson classification (p = 0.014) and tumor diameter > 50 mm (p = 0.039) remained significant independent variables for RFS in NSBM (**Table 4**).

**Table 4:** Univariate (logrank) and multivariate (logistic regression) analysis of factors associated with recurrence–free survival (RFS).

	Univariate analysis	Multivariate logistic regression analysis			
Parameter	p-value	Hazard Ratio	95% CI		p-value
**WHO grade**	0.0001	1.633	0.936	2.330	0.0001
**Venous sinus infiltration**	0.0010	0.354	-0.549	1.257	0.442
**Simpson classification**	0.0040	0.457	0.092	0.822	0.014
**Tumor diameter > 50 mm**	0.0250	0.767	0.038	1.497	0.039

#### 3.4.2 Overall survival (OS) and tumor-specific survival (TSS)

Overall, 49 patients died within the follow-up with a median OS of 51.3 months (0.5 - 153.2 months). For 19 of those patients (38.8%), the cause of death was tumor-related. Therefore, the median TSS was 53.7 months (3.1 – 153.2 months). The TSS Kaplan-Meier plot can be seen in **Figure 4B**. Eleven, six, and two patients died because of a WHO I, II, or III meningioma, resulting in a mortality of 4.3%, 13.6%, and 66.7% for the median follow-up of 87.0 months, respectively.

There was a significant association of WHO grading (p = 0.0001), Simpson classification (p = 0.0260) and venous sinus infiltration (p = 0.0140) with TSS in univariate log rank testing. All other characteristic (tumor diameter ≤/> 50 mm, age, sex, localization, MIB labeling index, bone infiltration) were not significantly correlated (p > 0.05). Multivariate logistic regression analysis of the three associated variables revealed that only WHO grading (p = 0.001) and Simpson classification (p = 0.031) remained significant independent variables for TSS (**Table 5**).

**Table 5:** Univariate (logrank) and multivariate (logistic regression) analysis of factors associated with tumor–specific survival (TSS).

	Univariate analysis	Multivariate logistic regression analysis			
Parameter	p-value	Hazard Ratio	95% CI		p-value
WHO grade	0.0001	1.477	0.614	2.341	0.0001
Venous sinus infiltration	0.0140	0.118	-1.105	1.341	0.850
Simpson classification	0.0260	0.535	0.048	10.210	0.031

## 4 Discussion

Meningiomas are mostly benign intracranial lesions in which surgical resection leads to durable tumor control (32, 35, 36). A recent study has demonstrated that low-risk meningiomas after GTR, the 10-year progression-free survival rate is 87.6% (37). Even in higher grade meningiomas, treated with surgical resection followed by adjuvant radiation treatment, a 10-year progression-free survival rate of 57.7% can be acomplished (38). In NSBM, recurrence rates range between 2.2% and 15% following gross total resection (39–41). Even after subtotal resection, long-term tumor control can be achieved with adjuvant radiation treatment (42) (38).As meningiomas may be considered to be a potentially curable disease, the actual clinical challenge for both patients and caregivers appears to arise from an entirely different aspect (32). According to a recent study, almost half of NSBM patients present with significant neurological symptoms (43) that stay unresolved over an extended period in 27% of the patients (27). In addition, meningioma patients frequently present with significant neurocognitive impairment (44), which persists in about 40% of the patients following surgical resection (45). Particularly patients with NSMB frequently experience partial and general seizures (26, 46), leading to antiepileptic drug treatment, which additionally causes compromised neurocognitive function (29, 47). These factors cause significantly impaired quality of life, even up to 10 years after initial diagnosis (48). Surgical resection may positively influence focal neurological impairment (24, 43), neurocognitive function (44), seizure frequency (26) and quality of life (30). Given NSBM’s specific biology, clinical dynamic, and symptomatology (11, 12, 25, 49), we attempted with our study to assess the functional recovery rates specifically in NSBM patients. Our data revealed that improvement rates of preoperative symptoms vary depending on the type of neurological deficit. Visual field deficits showed with 27.3%, the poorest improvement rate following surgery, which is in accordance with a recent study reporting only 16% recovery rate of visual field deficits in NSBM patients following surgical resection (24). Interestingly, studies summarizing focal neurological improvement rates in stroke patients also demonstrated a significantly worse recovery rate in visual field deficits (50, 51)compared to hemiparesis (52) or aphasia (53, 54). The most probable reason for this observation is founded on the optic system’s highly organized retinotopic and cortical functionality (53–55), causing the comparably low functional re–organization rates (55). In addition to location, tumor biology appears to significantly impact the surrounding brain and the resulting functional impairment. We detected a significantly higher frequency of larger edema in tumors with higher malignancy grade, which is in accordance with an earlier study reporting identical findings (56). Correspondingly, patients with higher-grade tumors in our study presented with a significantly higher frequency of focal neurological impairment and a poorer presurgical KPS, which aligns with a recent study reporting 56.1% of patients with atypical meningiomas showing a poor KPS (57). Most importantly, patients harboring higher-grade tumors displayed significantly worse improvement rates than benign lesions, highlighting the importance of tumor biology in this context. In our patient population, GTR is clearly superior compared to STR when analyzing recurrence–free survival. However, regarding neurological symptoms, only patients with seizures or visual field deficits showed a higher benefit from GTR over STR regarding symptom improvement, which indicates that most patients will benefit from surgical decompression, even if GTR cannot be achieved. Interestingly, GTR, compared to STR, carries a lower risk of developing a new or worsening of a pre–existing hemiparesis. When looking at the long-term functional outcome, poor resection grade was the only prognostic factor for new postoperative and permanent neurological deficit or decreased KPS score, which occurred in 7.3% of our patients.

## 5 Conclusion

Our study shows that surgical resection leads to long-term improvement of neurological impairment in the majority of patients with NSBM. However, location, tumor biology, and extent of resection are essential co-factors influencing neurological outcome.

## Data availability statement

The original contributions presented in the study are included in the article/supplementary material. Further inquiries can be directed to the corresponding author.

## Ethics statement

The studies involving human participants were reviewed and approved by Ethics committee of the University Regensburg Medical Center, protocol number 20-1799-101. The patients/participants provided their written informed consent to participate in this study.

## Author contributions

Conception and design (MP, TM, AB, and KR); analysis and interpretation of data (MP, TM, SS, AB, K-MS, N-OS, CD, and KR); manuscript draft and/or revision (TM, MP, K-MS, N-OS, and CD). All authors contributed to the article and approved the submitted version.

## Acknowledgments

We thank Michael Gerken (Tumor Center Regensburg, Institute of quality assurance and health services research, University of Regensburg, Regensburg, Germany) for his extensive support during clinical data acquisition.

## Conflict of interest

The authors declare that the research was conducted in the absence of any commercial or financial relationships that could be construed as a potential conflict of interest.

## Publisher’s note

All claims expressed in this article are solely those of the authors and do not necessarily represent those of their affiliated organizations, or those of the publisher, the editors and the reviewers. Any product that may be evaluated in this article, or claim that may be made by its manufacturer, is not guaranteed or endorsed by the publisher.

## Abbreviations

EOR, Extent of resection; FLAIR, Fluid-attenuated inversion recovery; GTR, Gross total resection; KPS, Karnofsky performance score; MRC-NPS, Medical Research Council - Neurological Performance Scale; NSBM, Non-skull base meningioma; RFS, Recurrence-free survival; SBM, Skull base meningioma; STR, Subtotal resection; TSS, Tumor-specific survival; vs., Versus.
